# The association of illness perceptions and God locus of health control with self-care behaviours in patients with type 2 diabetes in Saudi Arabia

**DOI:** 10.1080/21642850.2020.1805322

**Published:** 2020-08-13

**Authors:** Mohsen Alyami, Anna Serlachius, Ibrahim Mokhtar, Elizabeth Broadbent

**Affiliations:** a Department of Psychological Medicine, Faculty of Medical and Health Sciences, The University of Auckland, Auckland, New Zealand; b Ministry of Health, Diabetes and Endocrine Centre, King Khaled Hospital, Najran, Saudi Arabia

**Keywords:** Type 2 diabetes, illness perceptions, self-care behaviours, Saudi Arabia

## Abstract

**Objective:** To investigate the associations between illness perceptions, God locus of health control (GLHC) beliefs, and self-care behaviours in Saudi patients with type 2 diabetes (T2D).

**Design:** A cross-sectional study was conducted with 115 adults with T2D in a Saudi Arabian diabetes clinic. Illness perceptions, GLHC beliefs, and self-care behaviours were assessed using the Arabic versions of the Brief Illness Perception Questionnaire, God Locus of Health Control, and Summary of Diabetes Self-Care Activities. Logistic and linear regressions were conducted.

**Results:** Greater perceptions of personal control (OR = 2.07, *p =* .045) and diet effectiveness (OR =   2.73, *p =* .037) were associated with higher odds of adhering to general diet. Greater perceptions of diet effectiveness (*β* = 0.27, *p *= .034) and better understanding of T2D (*β* = 0.54, *p *< .001) were significant independent predictors of fruit and vegetables intake and exercise respectively. Patients with lower GLHC beliefs (OR =  4.40, *p = *.004) had higher odds of adhering to foot care than those with higher GLHC beliefs. Illness perceptions and GLHC beliefs did not predict adherence to a low-fat diet, self-monitoring of blood glucose, or not smoking.

**Conclusion:** Greater perceptions of personal control, coherence, diet effectiveness, and lower GLHC beliefs were associated with higher adherence to self-care behaviours in Saudi patients with T2D. Interventions designed to promote self-care behaviours in Saudi patients with T2D could focus on addressing these perceptions.

## Introduction

Type 2 diabetes (T2D) accounts for approximately 90% of all reported cases of diabetes (International Diabetes Federation, 2017). To improve health outcomes and quality of life, management guidelines recommend that individuals living with T2D engage in a range of self-care behaviours. These behaviours include following a healthy diet, being physically active, self-monitoring of blood glucose (SMBG), foot care, and smoking cessation (World Health Organization, 2016). Adherence to self-care behaviours is associated with better glycaemic control and improved quality of life in patients with T2D (Babazadeh et al., 2017; Colberg et al., 2010; Poolsup, Suksomboon, & Jiamsathit, 2008). However, research indicates that adherence to self-care behaviours among patients with T2D is suboptimal (Bonger, Shiferaw, & Tariku, 2018; da Rocha, Silva, & Cardoso, 2020; Mogre, Abanga, Tzelepis, Johnson, & Paul, 2017).

Non-adherence to self-care behaviours is a major concern in Saudi Arabia given the high prevalence of T2D (Robert & Al Dawish, 2020; Saad et al., 2018). More than 50% of patients report low levels of adherence to healthy eating, foot care, and SMBG behaviours (Al Johani, Kendall, & Snider, 2015) and physical activity (Ramadhan et al., 2019). These reports of non-adherence are consistent with findings in neighbouring Arab countries (Alhariri, Saghir, & Saghir, 2017; D’Souza et al., 2017).

The role of illness perceptions in the management of chronic illnesses including diabetes has been highlighted in the literature (Broadbent et al., 2015; Hagger, Koch, Chatzisarantis, & Orbell, 2017; Harvey & Lawson, 2009). The concept of illness perceptions is based on the Common Sense Model (CSM), which proposes that in response to a perceived health threat (illness), individuals create their own cognitive and emotional representations of the illness (known as illness perceptions), which influence how individuals cope with their illness (Diefenbach & Leventhal, 1996; Leventhal, Brissette, & Leventhal, 2003). Illness perceptions include perceptions about the illness’ identity (name and symptoms attributed to the illness), timeline (duration of the illness), consequences (impact of illness on individuals’ daily functioning), controllability (whether the illness can be cured or controlled via treatment), and perceived causes.

Based on the CSM, a scale was developed in which the controllability dimension was divided into personal control (individual’s own perceived control over management of the illness) and treatment control (whether treatment regimen can keep the illness under control) and timeline dimension was divided into timeline (acute/chronic) (duration of the illness) and cyclical timeline (day to day variability in symptoms) (Moss-Morris et al., 2002). Two subscales were also added, namely emotional response (affective response to the illness) and illness coherence (individual’s overall understanding of the illness). According to the CSM, emotional responses are processed in parallel to the cognitive representations, and together shape and guide individuals’ coping strategies including their adherence to self-care behaviours (Leventhal et al., 2003).

Previous research has shown that illness perceptions are associated with self-care behaviours in patients with T2D (Harvey & Lawson, 2009). For example, lower perceptions of consequences, illness identity, and emotional response, greater perceptions of acute timeline, personal control, treatment control, illness coherence, and diet effectiveness have been found to be associated with adherence to healthy dietary behaviours (Abubakari, Cousins, Thomas, Sharma, & Naderali, 2016; Broadbent, Donkin, & Stroh, 2011; French, Wade, & Farmer, 2013; Searle, Norman, Thompson, & Vedhara, 2007; van Puffelen et al., 2015). Greater perceptions of acute timeline, treatment control, personal control, coherence, and exercise effectiveness have been associated with being physically active (Broadbent et al., 2011; Searle et al., 2007; van Puffelen et al., 2015).

A more chronic and cyclical timeline, greater perceptions of illness identity, emotional response, personal control, treatment control, and coherence have been associated with foot care behaviour in patients with T2D (Abubakari et al., 2016; Indrayana, Guo, Lin, & Fang, 2019; Pereira, Pedras, & Ferreira, 2018; Vedhara et al., 2014). Evidence regarding the association between perceptions of consequences and adherence to foot care is mixed. Some research found that perceiving diabetes to have severe consequences on health status and daily functioning was associated with less frequent foot care (Abubakari et al., 2011; Indrayana et al., 2019) while other research found it was associated with more frequent foot care (Pereira et al., 2018). Additionally, there are mixed findings regarding perceptions of a chronic timeline, with one study finding it is associated with more frequent self-monitoring of blood glucose (Abubakari et al., 2016), and other research not finding this association (Abubakari et al., 2011; Hampson, Glasgow, & Foster, 1995).

Research has shown that the structure of illness perception domains is largely consistent across cultures, but the scores on those domains can vary between cultures (Broadbent, 2019). Evidence shows that distinct ethnic groups perceive diabetes differently (Abubakari et al., 2013; Alzubaidi, Mc Narmara, Kilmartin, Kilmartin, & Marriott, 2015; Barnes, Moss-Morris, & Kaufusi, 2004; Bean, Cundy, & Petrie, 2007). For example, Abubakari et al. (2013) found that, in comparison to White-British patients, Black-African and Black-Caribbean patients perceived T2D as a short-term condition with less consequences. Patients from the Pacific Islands perceived T2D as a condition with severe consequences, reported greater emotions, and attributed more symptoms to T2D than their European and South Asian counterparts (Bean et al., 2007).

Locus of health control, a multifaceted construct, can also affect adherence to treatment regimens in diabetes (Büyükkaya, Günüşen, Sürücü, & Koşar, 2016; O’Hea et al., 2009; Schlenk & Hart, 1984). This refers to the individuals’ perceptions of who or what controls their health within several domains including internal control, chance and powerful others (Wallston, Wallston, Kaplan, & Maides, 1976). Although existing illness perception questionnaires such as the IPQ (Weinman, Petrie, Moss-Morris, & Horne, 1996) and the B-IPQ (Broadbent, Petrie, Main, & Weinman, 2006) include items on control perceptions, these are only in relation to personal and treatment control. These perceptions are therefore distinct from God Locus of Health Control beliefs, which refer to the extent to which individuals believe God controls their health (Wallston et al., 1999). These perceptions are not diametrically opposed, it is possible that people hold beliefs that both they and God have some control over their health.

In the Middle East, including Saudi Arabia, religious beliefs such as fatalism are common (Al-Sahouri, Merrell, & Snelgrove, 2019; Al-Shahri, 2002). Fatalism can be defined as the belief that one’s life including health is predestined by God (Al-Shahri, 2002). Research in the Middle East and elsewhere has shown that patients with T2D who believe that God is in control of their health are less likely to adhere to their treatment regimens (Ahmedani, Peterson, Wells, Rand, & Williams, 2013; Albargawi, Snethen, Gannass, & Kelber, 2016; Jeragh-Alhaddad, Waheedi, Barber, & Brock, 2015).

It is unclear whether or not illness perceptions of T2D and GLHC beliefs in Saudi patients can predict the extent of their adherence to diabetes self-care behaviours. Therefore, the aim of this study was to investigate the relationships between illness perceptions and self-care behaviours in Saudi Arabia, including the GLHC beliefs. Findings from this study will help inform future interventions aimed at improving adherence to self-care behaviours among Saudi patients with T2D. Associations with medication adherence and glycaemic control have been reported in a separate paper (Alyami, Serlachius, Mokhtar, & Broadbent, 2019).

Informed by the literature, it was hypothesized that higher adherence to self-care behaviours would be associated with greater perceptions of personal control and treatment control, more chronic and cyclical timeline beliefs, and lower perceptions of consequences, illness identity, emotional response, and GLHC beliefs.

## Methods

### Sampling

The sample size calculation was based on a previous peer-reviewed paper that investigated the relationships between illness perceptions and self-care behaviours across three ethnic groups (Bean et al., 2007). Correlations ranged between .26 and −.37. A power calculation using G*Power software (Faul, Erdfelder, Lang, & Buchner, 2007) revealed that using the lower correlation (*r *= .26) with 80% power and .05 alpha, would require a total of 113 participants.

### Participants and procedure

Study participants were a convenience sample of Saudi patients with T2D recruited from an outpatient diabetes centre in Najran, Saudi Arabia. Patients were included if they (1) were 18 years of age or older (2) had a diagnosis of T2D for at least one year and (3) were taking oral medications and/or insulin. Patients were excluded if they were pregnant, feeling unwell on the day of recruitment, or not taking any medications for T2D.

Interested participants were invited and screened for eligibility in the clinic’s waiting areas. After written informed consent was obtained, patients filled in the questionnaire while waiting to be seen by their endocrinologist. Participants who were unable to read the questionnaire independently were assisted by the first author (who read the questionnaire items and recorded answers). Clinical information was extracted from patients’ medical records. Data collection occurred between Feb and March 2019. Patients received no compensation for participation. This study was approved by King Fahad Medical City Institutional Review Board (IRB 18-353E) and the Chairs of the University of Auckland Human Participants Ethics Committee.

### Measures

#### Self-care behaviours

Participants’ adherence to self-care behaviours was assessed using the Summary of Diabetes Self-Care Activities (SDSCA) (Toobert, Hampson, & Glasgow, 2000). The SDSCA consists of 11 items that assess the frequency of performing diabetes self-care behaviours over the preceding week. Assessed behaviours included adherence to general diet (2 items), specific diet (2 items), exercise (2 items), SMBG (2 items), and foot care (2 items). These 10 items are scored on an eight-point Likert scale (0–7). The mean number of days per week is used to score each subscale with higher scores corresponding to greater adherence to self-care behaviours. The last item is binary (yes/no response) and asked participants whether they had smoked a cigarette during the past seven days and was coded as 0 (yes) or 1 (no).

The Arabic version of the SDSCA has been found to be a reliable and valid measure of self-care behaviours among patients with T2D (Al Johani, Kendall, & Snider, 2016; Sukkarieh-Haraty & Howard, 2016). In the current study, the SDSCA subscales showed good to excellent internal consistency (general diet *α* = .99; exercise *α* = .75; SMBG *α* = .99; and foot care *α *= .67). The specific diet domain showed a low Cronbach alpha (*α* = .02), which was consistent with earlier research (Goh et al., 2016). Hence the specific diet items (fruit and vegetables and low-fat diet) were analysed separately as suggested by the original authors (Toobert et al., 2000). Responses on the general diet and foot care domains were negatively skewed and therefore scores were dichotomized based on the sample mean scores (scores higher than the mean were used to indicate better adherence).

#### Illness perceptions

Participants’ perceptions of T2D were measured using the Brief Illness Perception Questionnaire (B-IPQ) (Broadbent et al., 2006). The B-IPQ comprises nine items that assess patients’ cognitive and emotional perceptions of T2D including: consequences, timeline, (acute/chronic), personal control, treatment control, illness identity, concern, coherence, and emotional response. These items are scored using a scale from 0 to 10, with higher scores indicating stronger perceptions. The ninth item is an open-ended question which asks participants to list the three most likely causes of T2D. Patients’ responses on the open-ended question were categorized into four groups (psychosocial factors, behavioural factors, hereditary factors, and God’s). The psychometric properties of the B-IPQ have been reported elsewhere (Broadbent et al., 2006; Broadbent et al., 2015). The Arabic version of the B-IPQ has demonstrated good psychometric properties (Saarti, Jabbour, El Osta, Hajj, & Khabbaz, 2016).

Six items were added to the B-IPQ. One item asked patients about their perceptions of the cyclical nature of their T2D *(‘How much do your diabetes symptoms change from day to day?’*) and scored using a scale, where 0 = very stable and 10 = very changeable. This was based on the cyclical timeline dimension from the Revised Illness Perception Questionnaire (Moss-Morris et al., 2002), which previous research has shown to have negative associations with glycaemic control (Mc Sharry, Moss-Morris, & Kendrick, 2011) and adherence to self-care behaviours (Barnes et al., 2004; Nsereko et al., 2013). In addition, evidence has indicated that beliefs about the effectiveness of self-care behaviours are important (French et al., 2013; Glasgow, Hampson, Strycker, & Ruggiero, 1997). Hence, the remaining five items asked patients about their perceptions of the effectiveness of the recommended self-care behaviours (‘*How much do you think your [antidiabetic medications; insulin injections; healthy diet; weight management; and physical activity] can help your diabetes?*’). Format and scoring of these questions were similar to the original B-IPQ items, where 0 = not at all and 10 = extremely helpful. These items have been used previously with diabetes patients and shown to be associated with adherence (Broadbent et al., 2011).

#### God locus of health control

Participants’ God locus of health control beliefs were assessed using the God Locus of Health Control (GLHC) (Wallston et al., 1999). The GLHC comprises six items and is scored on a six-point Likert scale (1 = strongly disagree to 6 = strongly agree). Item scores are computed to give a total score ranging between 6 and 36, with higher total scores indicating a greater belief that God controls one’s health. The original GLHC English version has demonstrated adequate reliability and validity (Wallston et al., 1999). The Arabic version has been used in previous research and showed adequate internal consistency (*α* = .85) (Albargawi et al., 2016). In the current study, the GLHC exhibited excellent internal consistency (*α* = .97). Responses on the GLHC were positively skewed and hence scores were dichotomized into two categories (a score of 36 indicates higher GLHC beliefs while a score of ≤ 35 indicates lower GLHC beliefs). The majority of patients (*n *= 76, 66%) obtained the maximum total score while about a quarter of patients (*n* = 29, 25%) scored between 35 and 24, with the majority obtaining a score of 24.

### Demographic and clinical information

Information about patients’ age, sex, marital status, education, employment, and income were self-reported. Clinical information including BMI, duration of diabetes, type and number of prescribed medications, comorbidities, and diabetes-related complications were extracted from patients’ medical records.

### Statistical analysis

Data were examined for violations of statistical assumptions, and non-parametric tests were used due to deviations from normality for the dependent (adherence to self-care behaviours) and independent variables (demographic/clinical variables, B-IPQ and GLHC beliefs) (Field, 2009). Correlation analyses, logistic regression, chi square, and ANOVA were used to examine the relationships between variables. Spearman rho coefficient was used with two continuous variables (e.g. B-IPQ dimensions and eating fruit and vegetables), point-biserial coefficient was used with one continuous variable and one dichotomous variable (e.g. B-IPQ dimensions and foot care), and phi coefficient was used with two dichotomous variables (e.g. GLHC beliefs and foot care).

A series of logistic and multiple linear regressions were conducted to examine predictors of self-care behaviours. Variables that were significantly correlated with self-care behaviours were included in the predictive models, after careful inspection of multicollinearity. Inter-item correlations revealed high inter-correlations between the predictors. Collinearity statistics confirmed this observation for several predictors (VIF > 10 & tolerance statistics < .2). Therefore, several predictors were dropped from the regression model as recommended (Leech, Barrett, & Morgan, 2015). Analyses adjusted for age, sex, income and education, as these variables have been associated with self-care behaviours in previous research (Al Johani et al., 2015; Bonger et al., 2018; D’Souza et al., 2017; Mogre et al., 2017; Tan & Magarey, 2008), and other covariates specific to each outcome as indicated in the bivariate analyses. Patients with missing data were excluded from the analysis. In all tests, statistical significance was set at *p* < .05 (two-tailed). Data analysis was conducted using IBM SPSS Statistics version 25 (IBM Corp, 2017).

## Results

### Sample characteristics

In total, 173 patients were invited and screened for eligibility. Of those, 127 patients were eligible, and 115 patients agreed to participate (91% response rate). The mean age was 56 years (SD 12.43). More than half of patients were males (*n* = 67, 58%), married (*n* = 83, 72%), and earned 10,000 Saudi Riyal (SR) (≈ 2666 USD) or less a month (*n* = 70, 61%). Less than one third of patients had tertiary education (*n* = 28, 24%) and were employed (*n* = 33, 29%).

The mean time since diagnosis was 10.33 years (SD 7.60), and the mean BMI was 30.94 (SD 5.05), with the majority of patients either overweight (*n* = 37, 32%) or obese (*n* = 64, 56%). More than half of patients (*n* = 64, 56%) were prescribed both oral medications and insulin.

There were significant comorbidities (*n* = 75, 65%) and diabetes-related complications (*n* = 82, 71%), including coronary heart disease (*n* = 33, 44%), hypothyroidism (*n* = 24, 32%) and hypertension (*n* = 11, 15%), dyslipidaemia (*n* = 68, 83%), retinopathy (*n* = 22, 27%), and nephropathy (*n* = 7, 8%). Table 1 shows the mean scores for self-care behaviours, illness perceptions and God locus of health control.

**Table 1.** Means and standard deviations for study variables.

Variable	Mean (SD) or N (%)	Possible range (actual range)	*n*
SDSCA			
General diet	1.70 (2.10)	0–7 (0–7)	115
Fruit & vegetables	4.32 (1.41)	0–7 (1–7)	115
Low-fat diet	4.23 (1.67)	0–7 (1–7)	115
Exercise	3.91 (1.96)	0–7 (0–7)	115
SMBG	4.50 (2.07)	0–7 (0–7)	115
Foot Care	1.13 (1.49)	0–7 (0–5.5)	115
Smoking			115
Non-smokers	94 (81.7%)		
B-IPQ			
Consequences	6.95 (2.36)	0–10 (2–10)	115
Timeline (acute/chronic)	9.07 (1.05)	0–10 (6–10)	115
Personal control	5.49 (2.21)	0–10 (0–10)	115
Treatment control	6.69 (2.53)	0–10 (2–10)	115
Identity	6.95 (1.58)	0–10 (3–10)	115
Concerns	6.87 (2.53)	0–10 (0–10)	115
Coherence	3.68 (2.26)	0–10 (0–9)	115
Emotional response	6.13 (1.73)	0–10 (2–10)	115
Cyclical timeline	8.30 (1.65)	0–10 (2–10)	115
Oral medication effectiveness	6.88 (2.20)	0–10 (3–10)	115
Insulin effectiveness	6.85 (1.99)	0–10 (3–10)	62
Diet effectiveness	3.96 (2.74)	0–10 (0–10)	115
Weight management effectiveness	3.98 (2.77)	0–10 (0–10)	115
Exercise effectiveness	5.60 (2.59)	0–10 (0–10)	115
Perceived causes of T2D			72
Psychosocial factors	3 (4%)		
Behavioural factors	46 (64%)		
Hereditary factors	44 (61%)		
God’s will	19 (26%)		
GLHC beliefs			
Total score	33.55 (4.48)	6–36 (24–36)	105
High GLHC beliefs	76 (66%)		
Low GLHC beliefs	29 (25%)		

*Abbreviations*: SD, Standard Deviation; SDSCA, Summary of Diabetes Self-Care Activities; SMBG, Self-Monitoring of Blood Glucose; B-IPQ, Brief Illness Perception Questionnaire; GLHC, God Locus of Health Control.

### Associations between self-care behaviours and clinical/demographic variables

Younger age (*B *= −.77, *p *= .001) and shorter duration of T2D (*B* = −.06, *p *= .048) were associated with higher general diet scores. Those earning more than 10,000 SR (> 2666 USD) a month were more likely to adhere to general diet (*χ*^2 ^= 11.03, df = 1, *p *= .001) than those earning less.

Younger age (*r* = −.206, *p *= .027) and higher level of education *F* (3, 60) = 3.03, *p *= .036 were associated with eating the recommended fruit and vegetables intake. Tukey post-hoc analysis indicated that patients with tertiary education were significantly more likely to eat fruit and vegetables than those with a high school education (mean difference: 1.06, *p =* .029, 95% CI: 0.08, 2.04).

Younger age (*r* = −.202, *p *= .030) and employment status *F* (2, 72) = 3.69, *p *= .023 were associated with eating a low-fat diet. Tukey post-hoc analysis indicated that patients who were employed were significantly more likely to adhere to a low-fat diet than those who were unemployed (mean difference: 1.06, *p *= .017, 95% CI: 0.16, 1.96).

Younger age (*r* = −.250, *p *= .007) and employment status *F* (2, 112) = 4.60, *p *= .010 were associated with higher levels of exercise. Tukey post-hoc analysis indicated that patients who were employed were significantly more likely to exercise than those who were unemployed (mean difference: 1.33, *p *= .009, 95% CI: 0.27, 2.38).

Sex, marital status, and employment status were significantly correlated with smoking status. Patients who were male (*χ*^2 ^= 18.41, df = 1, *p *< .001), married (*χ*^2 ^= 4.29, df = 1, *p *= .038), and employed (*χ*^2 ^= 14.88, df = 2, *p* = .001) were significantly more likely to be smokers. None of the demographic or clinical variables showed no associations with foot care and SMBG.

### Associations between self-care behaviours, illness perceptions and God locus of health control beliefs

Spearman, point biserial and phi correlation coefficients showed significant relationships between illness perceptions, GLHC beliefs, and self-care behaviours (see Table 2).

**Table 2.** Correlations between study variables and self-care behaviours (*n* = 115).

	Correlation coefficients						
Variable	General diet^b^	Fruit & vegetables^a^	Low-fat diet^a^	Exercise^a^	SMBG^a^	Foot care^b^	Not smoking^b^
Consequences^a^	−.18	−.16	−.09	−.23*	−.07	.02	−.10
Timeline (acute/chronic) ^a^	−.05	−.13	.11	−.03	.13	−.09	−.12
Personal control^a^	.28**	.27**	.05	.28**	.04	.19*	.16
Treatment control^a^	.28**	.36**	.10	.28**	.07	.16	.16
Identity^a^	−.04	−.13	−.02	−.18	−.07	.09	−.19*
Concerns^a^	−.12	−.22*	−.16	−.20*	.001	.14	.12
Coherence^a^	.29**	.35*	.21*	.49**	.08	.15	.03
Emotional response^a^	−.01	−.18	−.04	−.16	−.07	.05	−.11
Cyclical timeline^a^	−.17	−.12	−.01	−.13	.08	−.01	−.12
Oral medication effectiveness^a^	.19*	.35**	.02	.20*	.22*	.15	.15
Insulin effectiveness^ac^	.18	.33**	.06	.15	.27*	.23	.05
Diet effectiveness^a^	.33**	.44**	.13	.18	.17	.14	.14
Weight management effectiveness^a^	.31**	.43**	.03	.17	.16	.17	.13
Exercise effectiveness^a^	.22*	.39**	.06	.19*	.21*	.13	.21*
GLHC beliefs^bd^	.05	−.18	.11	−.15	−.05	−.27**	.002

* *Note*:* ^a^Continuous variable; ^b^Dichotomous variable; ^c^62 total valid responses; ^d^105 total valid responses. We used Spearman rho coefficient with two continuous variables, Point-biserial coefficient with one continuous and one dichotomous variable, and Phi coefficient with two dichotomous variables.

*p < .05; **p < .01;

*Abbreviations:* SMBG, Self-Monitoring of Blood Glucose; GLHC, God Locus of Health Control

Chi square analysis indicated significant relationships between reported causal beliefs of T2D and adherence to general diet. Patients who adhered to general diet were less likely to perceive God’s will as a main cause of T2D (*χ*^2 ^= 8.06, df = 1, *p *= .005). There were no statistically significant differences in general diet scores between those who endorsed psychosocial factors (*χ*^2 ^= .197, df = 1, *p *= .657), behavioural factors (*χ*^2 ^= 2.31, df = 1, *p *= .129) or hereditary factors (*χ*^2 ^= .791, df = 1, *p *= .374) as a main cause of T2D and those who did not. There were also no statistically significant differences in foot care and smoking status with respect to the reported perceived causes of T2D (*p* > .05).

A One-Way ANOVA was conducted to examine the relationships between reported causal beliefs of T2D and eating the recommended fruit and vegetables intake, a low-fat diet, exercise, and SMBG. Patients who endorsed hereditary factors as a main cause of T2D were less likely to adhere to the recommended fruit and vegetables intake (mean 3.98, SD 1.55) than those who did not endorse hereditary factors (mean 4.79, SD 1.19) [F (1, 67) = 11.18, *p *= .022]. However, there were no statistically significant differences in low-fat diet, exercise, and SMBG scores between those who listed psychosocial, behavioural, hereditary factors or God’s will as a main cause of T2D and those who did not (*p *> .05).

### Predictors of self-care behaviours

Using logistic and multiple linear regressions, we examined which variables predicted adherence to recommended self-care behaviours. In model 1, we included demographic variables (age, sex, income, and education), based on earlier research (Al Johani et al., 2015; Bonger et al., 2018; D’Souza et al., 2017; Mogre et al., 2017; Tan & Magarey, 2008) and other covariates specific to each outcome. In model 2, we included variables that were significantly correlated with each of the self-care behaviours.

### Adherence to general diet

Using logistic regression, we examined which variables predicted adherence to a general diet. The first model with covariates including age, time since diagnosis, sex, income and education was significant (*χ*^2 ^= 25.05, df = 7, *p *= .001), with younger age (OR =  0.42, *p *= .014) and lower income (OR =  0.27 *p *= .004) associated with lower odds of adhering to a general diet. In model 2, perceptions of personal control, coherence, oral medication effectiveness, diet effectiveness, and exercise effectiveness were added, and the model was significant (*χ*^2 ^= 15.53, df = 5, *p*= .008). Lower income (OR =  0.19, *p *= .002) was associated with lower odds of adhering to a general diet, whereas greater perceptions of personal control (OR =  2.07, *p =* .045) and greater perceptions of diet effectiveness (OR =  2.73, *p =* .037) were associated with higher odds of adhering to a general diet (see Table 3).

**Table 3.** Logistic regression predicting adherence to general diet (*n* = 115)

Predictor	Model 1			Model 2		
	B (SE)	OR	95% CI	B (SE)	OR	95% CI
Constant	−0.09 (0.67)	0.92		0.23 (0.72)	1.25	
Age	−0.86* (0.35)	0.42	0.21, 0.84	−0.65 (0.39)	0.52	0.24, 1.12
Time since diagnosis	−0.34 (0.25)	0.71	0.44, 1.17	−0.47 (0.29)	0.62	0.36, 1.09
Sex (male)	0.45 (0.45)	1.57	0.65, 3.81	0.53 (0.50)	1.70	0.64, 4.54
Income (≤ 10,000 SR)	−1.30** (0.46)	0.27	0.11, 0.66	−1.67** (0.54)	0.19	0.07, 0.54
Education						
Tertiary	Reference group			Reference group		
Illiterate	0.85 (0.88)	2.33	0.41, 13.20	0.44 (0.96)	1.55	0.24, 10.07
Read & write	0.65 (0.71)	2.00	0.48, 7.76	0.76 (0.79)	2.14	0.45, 10.12
High school	−0.14 (0.67)	0.87	0.24, 3.21	−0.37 (0.72)	0.69	0.17, 2.81
Personal control				0.73* (0.38)	2.07	0.99, 4.34
Coherence				0.03 (0.34)	1.03	0.53, 2.02
Oral medication effectiveness				−0.37 (0.37)	0.69	0.33, 1.43
Diet effectiveness				1.00* (0.48)	2.73	1.06, 7.02
Exercise effectiveness				−0.49 (0.45)	0.62	0.26, 1.47
*Hosmer & Lemeshow R^2^*	.590			.146		
*Nagelkerke R^2^*	.262			.398		

Note*:* Two predictors (treatment control and weight management effectiveness) were excluded due to multicollinearity. *p < .05; **p < .01.

*Abbreviations:* B, Beta; SE, Standard Error; OR, Odds Ratio; CI, Confidence Interval; SR, Saudi Riyal.

### Adherence to foot care

Using logistic regression, we examined which variables predicted adherence to foot care. Model 1 with the covariates, including age, sex, income, and education, was not significant (*χ*^2 ^= 11.46, df = 6, *p *= .075). In model 2, perceptions of personal control and GLHC beliefs were added, and the model was significant (*χ*^2 ^= 11.47, df = 2, *p *= .003), with lower education level (illiterate) (OR =  12.39, *p =* .011) and lower GLHC beliefs (OR =  4.40, *p = *.004) associated with higher odds of adhering to foot care (see Table 4).

**Table 4.** Logistic regression predicting foot care (*n* = 105).

Predictor	Model 1			Model 2		
	B (SE)	OR	95% CI	B (SE)	OR	95% CI
Constant	−0.91 (0.67)	0.40		−1.25 (0.73)	0.29	
Age	−0.54 (0.32)	0.58	0.32, 1.08	−0.48 (0.34)	0.62	0.32, 1.20
Sex (male)	−0.12 (0.44)	0.89	0.38, 2.10	−0.34 (0.47)	0.71	0.28, 1.79
Income (≤ 10,000 SR)	−0.64 (0.46)	0.53	0.22, 1.30	−0.67 (0.49)	0.51	0.20, 1.34
Education						
Tertiary	Reference group			Reference group		
Illiterate	2.32* (0.93)	10.19	1.64, 63.24	2.52* (0.99)	12.39	1.77, 87.03
Read & write	1.08 (0.73)	2.94	0.71, 12.21	1.04 (0.78)	2.83	0.62, 13.00
High school	0.27 (0.70)	1.32	0.34, 5.15	0.28 (0.75)	1.32	0.31, 5.68
Personal control				0.25 (0.23)	1.29	0.83, 2.01
Lower GLHC beliefs				1.48** (0.52)	4.40	1.60, 12.11
*Hosmer & Lemeshow R^2^*	.341			.418		
*Nagelkerke R^2^*	.139			.265		

Note*:* *p < .05; **p < .01.

*Abbreviations*: B, Beta; SE, Standard Error; OR, Odds Ratio; CI, Confidence Interval; SR, Saudi Riyal; GLHC, God Locus of Health Control.

### Adherence to the recommended fruit and vegetables intake

Using linear regression, we examined which variables predicted adherence to the recommended fruit and vegetables intake. Model 1 with the covariates, including age, sex, income, and education, was significant (*F* (5, 81) = 3.50, *p *= .006, R^2 ^= .178). Earning more than 10,000 SR (high income) (*β* = 0.29*, p* = .008) predicted eating more fruit and vegetables, whereas having a lower education level (being able to read and write only) (*β *= −0.32, *p *= .008) predicted eating less fruit and vegetables. In Model 2, perceptions of personal control, coherence, concerns, and diet effectiveness were added. Model 2 was also significant (*F* (9, 77) = 4.91, *p *< .001, R^2 ^= .365). The addition of illness perception domains into the model explained an additional 18.7% of the total variance. Earning high income (*β* = 0.29, *p *= .005), having a lower education level (*β *= −0.29, *p *= .009), and higher perceptions of diet effectiveness (*β* = 0.27, *p *= .034) were significant independent predictors of fruit and vegetables intake in the regression model (see Table 5).

**Table 5.** Linear regression predicting eating the recommended fruit and vegetables intake (*n* = 115).

Predictor	Model 1			Model 2		
	B (SE)	*β*	95% CI	B (SE)	*β*	95% CI
Constant	4.40 (0.27)		3.86, 4.94	4.38 (0.25)		3.88, 4.87
Age	−0.20 (0.23)	−0.12	−0.65, 0.26	0.10 (0.22)	0.06	−0.34, 0.54
Sex (male)	−0.16 (0.32)	−0.06	−0.80, 0.47	−0.12 (0.29)	−0.04	−0.70, 0.46
Income (>10,000 SR)	0.83** (0.31)	0.29	0.22, 1.43	0.82** (0.28)	0.29	0.26, 1.38
Education						
Tertiary	Reference group			Reference group		
Read & write	−0.98** (0.36)	−0.32	−1.70, −0.26	−0.88** (0.33)	−0.29	−1.54, −0.23
High school	−0.24 (0.44)	−0.08	−1.13, 0.64	−0.13 (0.41)	−0.04	−0.94, 0.68
Personal control				−0.02 (0.19)	−0.02	−0.39, 0.35
Coherence				0.29 (0.18)	0.21	−0.06, 0.64
Concerns				−0.14 (0.16)	−0.10	−0.47, 0.19
Diet effectiveness				0.36* (0.17)	0.27	0.03, 0.69
R²	0.18			0.37		
Adjusted R²	0.13			0.29		

Note*:* Five predictors (treatment control, oral medication effectiveness, insulin effectiveness, weight management effectiveness, and exercise effectiveness) were excluded due to multicollinearity. *p < .05; **p < .01.

*Abbreviations*: B, unstandardized beta coefficient; SE, Standard Error; *β* standardized beta coefficients; CI, Confidence Interval; SR, Saudi Riyal.

### Exercise

Using linear regression, we examined which variables predicted adherence to exercise. Model 1 with the covariates, including age, sex, employment, income, and education, was not significant (*F* (6, 80) = 1.78, *p *= .113, R^2 ^= .118). Model 2 included perceptions of consequences, personal control, coherence, concerns and exercise effectiveness and was significant (*F* (11, 75) = 3.62, *p *< .001, R^2 ^= .347). The addition of illness perception variables explained an additional 22.9% of the total variance in exercise. Higher perceptions of coherence (*β* = 0.54, *p *< .001) were a significant independent predictor of exercise in the regression model (see Table 6).

**Table 6.** Linear regression predicting exercise (*n* = 115).

Predictor	Model 1			Model 2		
	B (SE)	*β*	95% CI	B (SE)	*β*	95% CI
Constant	4.13 (0.42)		3.29, 4.20	3.97 (0.38)		3.20, 4.73
Age	−0.62 (0.36)	−0.28	−1.33, 0.09	−0.07 (0.34)	−0.03	−0.76, 0.62
Sex (male)	−0.70 (0.48)	−0.18	−1.66, 0.27	−0.37 (0.44)	−0.10	−1.26, 0.52
Employment						
Retired	Reference group			Reference group		
Unemployed	0.59 (0.57)	0.15	−0.56, 1.73	0.87 (0.52)	0.23	−0.17, 1.92
Income (> 10,000 SR)	−0.24 (0.43)	−0.06	−1.10, 0.61	0.03 (0.38)	0.01	−0.74, 0.79
Education						
Tertiary	Reference group			Reference group		
Read & write	0.06 (0.50)	0.01	−0.94, 1.06	−0.08 (0.46)	−0.02	−1.00, 0.83
High school	−0.33 (0.65)	−0.08	−1.62, 0.96	−0.68 (0.59)	−0.17	−1.84, 0.49
Consequences				−0.09 (0.30)	−0.05	−0.70, 0.51
Personal control				0.09 (0.29)	0.05	−0.49, 066
Coherence				1.01**(0.23)	0.54	0.54, 1.47
Concerns				0.17 (0.25)	0.09	−0.33, 0.68
Exercise effectiveness				−0.13 (0.20)	−0.07	−0.54, 0.28
R²	0.12			0.35		
Adjusted R²	0.05			0.25		

Note*:* Two predictors (treatment control and oral medication effectiveness) were excluded due to multicollinearity. **p < .01.

*Abbreviations*: B, unstandardized beta coefficient; SE, Standard Error; *β* standardized beta coefficients; CI, Confidence Interval; SR, Saudi Riyal.

### Adherence to a low-fat diet, not smoking and SMBG

Neither the demographic variables (age, sex, marital status, employment, income, and education) nor patients’ illness perceptions were associated with eating a low-fat diet, not smoking, or SMBG in the fully adjusted predictive models (see Supplementary Tables 7–9).

## Discussion

This is the first study to examine the role of illness perceptions and God locus of health control in self-care behaviours among Saudi patients with T2D. Illness perceptions were associated with adherence to a general diet, fruit and vegetable intake and exercise, while God locus of health control beliefs were associated with foot care. These findings support previous research in other populations and extend the application of the CSM to the Saudi population.

Consistent with previous research (Al Johani et al., 2015; da Rocha et al., 2020; Saad et al., 2018), adherence to self-care behaviours was low. A likely explanation, though not tested in the present study, could be patients’ insufficient knowledge about diabetes, the importance of self-care in diabetes, and the lack of focused and effective education about diabetes management. A number of studies have documented the lack of knowledge about T2D management in Saudi patients with T2D (Al-Aboudi, Hassali, & Shafie, 2016; Alhaik, Anshasi, Alkhawaldeh, Soh, & Naji, 2019; Saad et al., 2018), and we know that knowledge is a strong predictor of adherence self-care behaviours (Kugbey, Oppong Asante, & Adulai, 2017). Improving patients’ understanding of diabetes through targeted education can increase engagement in self-care behaviours (Al Slamah, Nicholl, Alslail, & Melville, 2017; Mohammad & Khresheh, 2018) and improve glycaemic control (Chrvala, Sherr, & Lipman, 2016; Tanash, Fitzsimons, Coates, & Deaton, 2017).

Adherence varied across the different kinds of self-care behaviours assessed in the present study. Patients were most adherent to SMBG, recommended intake of fruit and vegetables, eating a low-fat diet, and exercise. Foot care and general healthy eating behaviours were the least performed behaviours during the preceding seven days. Although this finding is in concordance with previous research, it is different in terms of which self-care behaviours were most practiced. For example, Saad and colleagues found that foot care was the most practiced self-care behaviour while exercise and SMBG were the least practiced behaviours among Saudi patients with T2D (Saad et al., 2018). The differences may be related to the fact that our participants were younger and had been living with T2D for a shorter time. Our analysis demonstrated that younger age was significantly correlated with better adherence to healthy eating behaviours and exercise. Longer duration of T2D was associated with poorer adherence to healthy eating behaviours in our study, which supports previous research linking longer duration of diabetes to lower adherence to self-care behaviours and suboptimal glycaemic control (Adwan & Najjar, 2013; Ko et al., 2012).

Overall, patients perceived T2D as a chronic and cyclical condition with severe consequences. Patients also reported low coherence of T2D and had low beliefs in the effectiveness of diet and weight management in controlling their T2D. These perceptions may have been influenced by a number of factors including patients’ demographic and clinical characteristics. Our sample had more male patients than female patients, and the overwhelming majority (71%) had at least one complication. Earlier research has shown sex-related (Boonsatean, Carlsson, Dychawy Rosner, & Östman, 2018) and complication-related differences in illness perceptions in patients with T2D (Searle et al., 2008; van Puffelen et al., 2015). For example, men with T2D reported greater beliefs that the treatment regimen can control their diabetes than women, while women perceived T2D to have a greater cyclical timeline with severe consequences than men (Boonsatean et al., 2018). Patients with diabetes-related complications had higher perceptions of consequences, illness identity, cyclical timeline, and emotional response and lower perceptions of personal and treatment control than patients without complications (van Puffelen et al., 2015).

Greater perceptions of personal control and diet effectiveness predicted improved adherence to the recommended healthy eating behaviours (general diet and recommended fruit and vegetables intake), which supports previous findings (Broadbent et al., 2011; van Puffelen et al., 2015). Patients who perceive they can control T2D using diet management are likely to engage in healthy eating behaviours. However, several studies have shown that perceptions of fewer consequences, lower illness identity, and lower emotional response also played a role in adherence to healthy eating behaviours (Broadbent et al., 2011; French et al., 2013).

Consistent with previous studies (Broadbent et al., 2011; French et al., 2013; van Puffelen et al., 2015), adherence to exercise was associated with greater personal control, treatment control, coherence, and exercise effectiveness. However, only higher perceptions of coherence were a significant independent predictor in the regression model indicating that the more patients understand T2D the more likely they adhere to exercise recommendations. Previous research has shown that perceiving exercise as helpful in controlling diabetes was associated with higher adherence to exercise regimens (Broadbent et al., 2011; French et al., 2013).

Although perceptions of oral medication, insulin, and exercise effectiveness in controlling T2D were positively associated with more frequent SMBG, these perceptions did not add a unique value to the explained variance after controlling for demographic variables. This finding supports previous research showing that the associations between illness perception domains and SMBG have been the least consistent in the literature (Harvey & Lawson, 2009). Some research found that perceptions of chronic timeline of diabetes were significantly correlated with more frequent SMBG (Abubakari et al., 2016), although other research did not (Hampson et al., 1995).

Perceiving God’s will and hereditary factors as main causes of T2D was associated with poor adherence to healthy eating behaviours. Previous research has shown that perceiving that T2D was caused by smoking, unhealthy eating, and poor medical care was associated with poor adherence to healthy eating behaviours, which differs to our findings (Barnes et al., 2004; van Puffelen et al., 2015). There were no associations between causal beliefs and adherence to foot care, exercise, SMBG, and smoking status in our sample.

God locus of health control beliefs were very strong in this sample, which is consistent with research with Muslim patients with diabetes (Al-Sahouri et al., 2019; Albargawi et al., 2016; Jeragh-Alhaddad et al., 2015). These fatalistic beliefs may contribute to the reported low adherence to self-care behaviours, as patients may not place as much value on their own abilities in the management of their T2D. Patients who believed God was in less control of their T2D, as opposed to total control, were more likely to adhere to foot care. This finding supports earlier research which showed that patients who believed God was solely responsible for whether their diabetes got better or worse were less likely to adhere to treatment regimens including medications and healthy eating behaviours (Albargawi et al., 2016; Jeragh-Alhaddad et al., 2015; Nabolsi & Carson, 2011). However, while it is true that God-centred locus of health control is consistent with the Islamic faith (Yosef, 2008), Islamic teachings also encourage believers to be responsible, engage in preventive behaviours, and seek professional help.

### Limitations and future directions

Our findings should be interpreted with caution in light of some limitations. First, this was a cross-sectional study and hence causality cannot be inferred. Second, all of our participants were recruited from a single diabetes outpatient clinic, which limits the generalizability of our findings. Future research should consider recruiting participants from multiple diabetes clinics and employing a longitudinal design to further understand how illness perceptions and changes in illness perceptions over time affect adherence to self-care behaviours among Saudi patients. Finally, two of the regression models had 12 predictors with 115 cases so had slightly fewer cases than the rule of thumb of 10 cases per predictor (Field, 2009).

### Implications

Our findings highlight the potential of examining patients’ illness perceptions, particularly perceptions of personal control, coherence, and diet effectiveness, in order to promote adherence to self-care behaviours. Existing interventions aimed at changing illness perceptions have shown promising results in patients with diabetes, including increased adherence to general diet and exercise (Keogh et al., 2011), more frequent blood glucose monitoring (Steed et al., 2005), and reduced smoking (Davies et al., 2008). Health professionals and educators working with Saudi patients with T2D should be aware of the associations between illness perceptions and adherence to recommended self-care behaviours. Providing information to patients alone is not enough to improve adherence and it is suggested that professionals administer the B-IPQ to patients with poorly controlled T2D to assess their perceptions as part of clinical care. If the B-IPQ indicates that patients have maladaptive perceptions, then health professionals and educators can target individuals’ beliefs to encourage more accurate and adaptive perceptions.

## Conclusion

Illness perceptions including personal control, coherence, and diet effectiveness, and God locus of health control beliefs are associated with patients’ adherence to self-care behaviours among Saudi patients with T2D. Interventions designed to promote self-care behaviours in Saudi patients with T2D should focus on addressing these perceptions.

## Supplementary Material

Supplemental Material

## Data Availability

The data that support the findings of this study are available upon reasonable request from the corresponding author, [EB]. The data are not publicly available due to containing information that could compromise the privacy of research participants.
