# Supplementary material for: The association of illness perceptions and God locus of health control with self-care behaviours in patients with type 2 diabetes in Saudi Arabia
Source: Health Psychol Behav Med. 2020 Aug 13;8(1):329–48. doi: 10.1080/21642850.2020.1805322 (PMC8114366; doi:10.1080/21642850.2020.1805322)
Supplement: Supplemental Material [file RHPB_A_1805322_SM1198.docx]

**Supplementary Tables**

Table 7. Linear regression predicting eating a low-fat diet (n= 115)

| Predictor | Model 1 | | | Model 2 | | |
| --- | --- | --- | --- | --- | --- | --- |
|  | B (SE) | β | 95% CI | B (SE) | β | 95% CI |
| Constant | 4.62 (0.40) |  | 3.83, 5.41 | 4.59 (0.40) |  | 3.80, 5.39 |
| Age | -0.54 (0.34) | -0.27 | -1.21, 0.13 | -0.40 (0.36) | -0.19 | -1.11, 0.32 |
| Sex (male) | -0.85 (0.45) | -0.24 | -1.75, 0.05 | -0.82 (0.46) | -0.23 | -1.73, 0.09 |
| Employment |  |  |  |  |  |  |
| Retired | Reference group | | | Reference group | | |
| Unemployed | -0.25 (0.54) | -0.01 | -1.10, 1.05 | 0.08 (0.55) | 0.02 | -1.01, 1.16 |
| Income (> 10000 SR) | -0.09 (0.40) | -0.03 | -0.89, 0.70 | -0.12 (0.41) | -0.03 | -0.93, 0.69 |
| Education |  |  |  |  |  |  |
| Tertiary | Reference group | | | Reference group | | |
| Read & write | -0.31 (0.47) | -0.08 | -1.25, 0.63 | -0.26 (0.47) | -0.07 | -1.21, 0.68 |
| High school | 0.10 (0.61) | 0.03 | -1.10, 1.31 | 0.13 (0.61) | 0.04 | -1.09, 1.35 |
| Coherence |  |  |  | 0.03 (0.24) | 0.02 | -0.44, 0.50 |
| Diet effectiveness |  |  |  | 0.23 (0.22) | 0.14 | -0.19, 0.66 |
| R² | 0.10 |  |  | 0.12 |  |  |
| Adjusted R² | 0.32 |  |  | 0.30 |  |  |

*Abbreviations*: B, unstandardized beta coefficient; SE, Standard Error; β standardized beta coefficients; CI, Confidence Interval; SR, Saudi Riyal

Table 8. Logistic regression predicting not smoking (n= 115)

| Predictor | Model 1 | | | Model 2 | | |
| --- | --- | --- | --- | --- | --- | --- |
|  | B (SE) | OR | 95% CI | B (SE) | OR | 95% CI |
| Constant | 2.72 (0.97) | 15.07 |  | 2.99 (1.00) | 19.81 |  |
| Age | -0.21 (0.37) | 0.81 | 0.40, 1.66 | -0.07 (0.39) | 0.93 | 0.43, 2.02 |
| Marital status (married) | -1.42 (0.81) | 0.24 | 0.05, 1.18 | -1.44 (0.82) | 0.24 | 0.05, 1.18 |
| Income (≤ 10000 SR) | -0.85 (0.58) | 0.43 | 0.14, 1.35 | -0.96 (0.62) | 0.38 | 0.11, 1.29 |
| Education |  |  |  |  |  |  |
| Tertiary | Reference group | | | Reference group | | |
| Illiterate | 1.66 (1.12) | 5.23 | 0.59, 46.60 | 1.70 (1.19) | 5.42 | 0.52, 56.24 |
| Read & write | 0.47 (0.78) | 1.60 | 0.34, 7.49 | 0.50 (0.85) | 1.65 | 0.31, 8.68 |
| High school | 0.15 (0.73) | 1.16 | 0.28, 4.84 | 0.06 (0.75) | 1.06 | 0.24, 4.63 |
| Illness identity |  |  |  | -0.52 (0.30) | 0.60 | 0.34, 1.06 |
| Exercise effectiveness |  |  |  | 0.40 (0.27) | 1.49 | 0.88, 2.52 |
| *Hosmer & Lemeshow R^2^* | .731 |  |  | .533 |  |  |
| *Nagelkerke R^2^* | .134 |  |  | .230 |  |  |

*Abbreviations*: B, Beta; SE, Standard Error; OR, Odds Ratio; CI, Confidence Interval; SR, Saudi Riyal

Table 9. Linear regression predicting SMBG (n= 115)

| Predictor | Model 1 | | | Model 2 | | |
| --- | --- | --- | --- | --- | --- | --- |
|  | B (SE) | β | 95% CI | B (SE) | β | 95% CI |
| Constant | 4.49 (0.43) |  | 3.63, 5.36 | 4.13 (1.01) |  | 2.13, 6.14 |
| Age | -0.21 (0.36) | -0.09 | -0.94, 0.51 | -0.09 (0.37) | -0.04 | -0.82, 0.64 |
| Sex (male) | -0.02 (0.51) | -0.01 | -1.03, 0.99 | -0.02 (0.51) | -0.01 | -1.03, 0.99 |
| Income (> 10000 SR) | 0.37 (0.49) | 0.09 | -0.59, 1.34 | 0.33 (0.48) | 0.08 | -0.63, 1.28 |
| Education |  |  |  |  |  |  |
| Tertiary | Reference group | | | Reference group | | |
| Read & write | -0.19 (0.58) | -0.04 | -1.39, 0.95 | -0.05 (0.58) | -0.01 | -1.19, 1.10 |
| High school | -0.22 (0.71) | -0.05 | -1.63, 1.18 | -0.03 (0.70) | -0.01 | -1.43, 1.37 |
| Oral medication effectiveness |  |  |  | 0.36 (0.29) | 0.18 | -0.22, 0.94 |
| Exercise effectiveness |  |  |  | 0.09 (0.29) | 0.05 | -0.48, 0.66 |
| R² | 0.02 |  |  | 0.06 |  |  |
| Adjusted R² | -0.05 |  |  | -0.02 |  |  |

*Note:* One predictor (insulin effectiveness) was excluded due to multicollinearity.

*Abbreviations*: SMBG, self-monitoring of blood glucose; B, unstandardized beta coefficient; SE, Standard Error; β standardized beta coefficients; CI, Confidence Interval; SR, Saudi Riyal
